# A modelled economic evaluation comparing atomoxetine with methylphenidate in the treatment of children with attention-deficit/hyperactivity disorder in Spain

**DOI:** 10.1186/1471-244X-9-15

**Published:** 2009-04-14

**Authors:** Jihyung Hong, Tatiana Dilla, Jorge Arellano

**Affiliations:** 1 LSE Health, London School of Economics, London, UK; 2 Eli Lilly and Company, Windlesham, UK; 3 Eli Lilly and Company, S.A, Alcobendas (Madrid), Spain

## Abstract

**Background:**

Attention Deficit/Hyperactivity Disorder (ADHD) is a neurobehavioural disorder, affecting 3–6% of school age children and adolescents in Spain. Methylphenidate (MPH), a mild stimulant, had long been the only approved medication available for ADHD children in Spain. Atomoxetine is a non-stimulant alternative in the treatment of ADHD with once-a-day oral dosing. This study aims to estimate the cost-effectiveness of atomoxetine compared to MPH. In addition, atomoxetine is compared to 'no medication' for patient populations who are ineligible for MPH (i.e. having stimulant-failure experience or co-morbidities precluding stimulant medication).

**Methods:**

An economic model with Markov processes was developed to estimate the costs and benefits of atomoxetine versus either MPH or 'no medication'. The incremental cost per quality-adjusted life-year (QALY) was calculated for atomoxetine relative to the comparators. The Markov process incorporated 14 health states, representing a range of outcomes associated with treatment options. Utility values were obtained from the utility valuation survey of 83 parents of children with ADHD. The clinical data were based on a thorough review of controlled clinical trials and other clinical literature, and validated by international experts. Costs and outcomes were estimated using Monte Carlo simulation over a 1-year duration, with costs estimated from the perspective of the National Health Service in Spain.

**Results:**

For stimulant-naïve patients without contra-indications to stimulants, the incremental costs per QALY gained for atomoxetine were € 34 308 (compared to an immediate-release MPH) and € 24 310 (compared to an extended-release MPH). For those patients who have stimulant-failure experience or contra-indications to stimulants, the incremental costs per QALY gained of atomoxetine compared to 'no medication' were € 23 820 and € 23 323, respectively.

**Conclusion:**

The economic evaluation showed that atomoxetine is an effective alternative across a range of ADHD populations and offers value-for money in the treatment of ADHD.

## Background

Attention Deficit/Hyperactivity Disorder (ADHD) is a neurobehavioural disorder and one of the most prevalent chronic health problems affecting school-age children [1], representing a costly major public health problem [2]. It begins early in childhood and persists throughout adolescence and well into adulthood in the majority of cases [3,4]. Affected children commonly exhibit disruptive behaviour in the classroom, underachieve academically and tend to have conflictive relations with family members and peers [5]. ADHD is also frequently associated with co-morbidities such as learning disorders, tics, anxiety and conduct disorders [6-8]. Without effective treatments, difficulties associated with the disorder may have long-term negative consequences such as difficulties in employment or in forming a good relationship, as well as the risk of substance abuse, crime and accidental injury [9-14]. In Spain, the estimated prevalence of ADHD among school-aged children is around 3–6% [15-17]

Multi-disciplinary approach to the management of ADHD is often suggested, in which medication may be an integral part when remedial measures alone prove insufficient [18]. In Spain, medications licensed for the treatment of ADHD include methylphenidate hydrochloride (MPH) and atomoxetine. MPH, which is mainly available as either immediate-release (IR) or an extended-release (XR) formulation, has been by far the most widely used medication for ADHD worldwide. Given that MPH is a stimulant medication however, it may have abuse risk or produce variations in mood state, sleep disorder or increase in tic severity [19]. MPH is thus contraindicated in patients with severe depression, marked anxiety, tics, a family history or diagnosis of Tourette's syndrome, known drug dependence or a history of drug dependence or alcoholism [20]. Treatment guidelines for some of these contra-indications may have not been followed strictly in the past due to the lack of alternative medication options. Atomoxetine, newly introduced in the Spanish market, is an alternative to stimulants in the treatment of ADHD with once-a-day oral dosing [20]. There is consistent evidence that atomoxetine is superior to placebo while no clear differences have been found between atomoxetine and MPH on the grounds of clinical efficacy in terms of standard measures of ADHD symptom control [21] though there has been increasing evidence in favour of MPH [22]. However, atomoxetine may have longer lasting effects compared to MPH. Results of a placebo-controlled trial suggests that, among those patients who respond to atomoxetine, a single dose each morning provides a lasting effect through to the following morning, provided that the medication is taken on a regular daily basis [23]. In contrast, the duration of efficacy of MPH may be more limited. A single dose of XR-MPH, or three repeated doses of IR-MPH, would provide about 12 hours of therapeutic coverage [24-26]. In view of their pharmacokinetic and pharmacodynamic characteristics as well as usual dose regimens, it is as such unlikely that these drugs would provide therapeutic coverage through the night or at the time of waking.

The objective of the present study was to estimate the cost-effectiveness of atomoxetine compared to MPH in the treatment of children with ADHD. As atomoxetine may offer a viable alternative for a substantial proportion of ADHD children who were ineligible for MPH due to a history of stimulant treatment failure and/or co-morbidities contra-indicated to simulants, atomoxetine treatment for these children was also compared to 'no medication' since they would usually have no alternative medication options otherwise. The present economic model adapted the UK model [27] and was modified to compare the costs and benefits of atomoxetine to that of either MPH or 'no medication' in the Spanish context.

## Methods

### Patient population

In recognition that i) children with ADHD are frequently co-diagnosed with one or more co-morbidities [28] some of which are contra-indicated for medication with stimulants [29] and ii) a patient's past stimulant history is a determining factor in clinical outcomes [30,31], patients in the evaluation are segmented into three mutually exclusive patient groups, according to history of stimulant treatment failure and contra-indication status.

• Stimulant-naïve patients without contra-indications to stimulants (population 1): These are patients with no history of pharmacotherapy use and no contra-indications to stimulants

• Stimulant-failed patients without contra-indications to stimulants (population 2): These are patients who have previously (prior to entry into the model) been medicated with MPH but have failed due to lack of efficacy or intolerable side effects

• Stimulant-naïve patients with contra-indications to stimulants (population 3): These patients have no history of pharmacotherapy use but are precluded from using stimulant therapies due to a pre-existing contra-indicated condition(s) – including severe depression, marked anxiety, tics, a family history or diagnosis of Tourette's syndrome, known drug dependence or a history of drug dependence or alcoholism [29]

Patients who were currently successfully being treated with MPH were excluded from the analysis because it was assumed that these patients were unlikely to switch medication.

### Treatments and comparators

The approach used to calculate the cost-effectiveness of atomoxetine is based on current treatment available to each of the patient groups hence reflecting the likely impact on cost and outcomes in real practice for each of them.

Atomoxetine was compared to MPH (IR-MPH and XR-MPH respectively) as a first-line therapy in population 1. Patients could switch from atomoxetine to MPH and *vice versa *as a second-line treatment when the first-line treatment failed. Subsequently they could stop all therapies upon failure of second-line therapy and remain on 'no medication' until the end of the model. For those patient groups (population 2 and 3) who are ineligible for MPH treatment, atomoxetine was compared to 'no medication'. Patients treated with atomoxetine could discontinue the medication upon failure of therapy and remain un-medicated until the end of the model while patients in the 'no medication' arm would remain un-medicated throughout the model.

### Model structure

The UK economic model [27] was adapted and re-constructed using TreeAge Pro software [32] to calculate and compare the costs and benefits of atomoxetine to that of either MPH or 'no medication' in the Spanish context. The economic evaluation employed a cost-utility analysis to calculate the incremental cost per quality-adjusted life-year (QALY) gained by atomoxetine compared with the treatment options available in Spain.

The model employed a Monte Carlo simulation, whereby a single patient was followed through the Markov process in monthly cycles over a period of one year. It was deemed inappropriate to extend the model beyond the timeframe covered by the available clinical data. Instead, it was implicitly assumed that there are no differences in health benefits between the medications in the longer term. Costs and outcomes were accumulated as the patient advanced through the cycles and 20,000 simulations were performed for each patient population to establish the mean costs and outcomes across all possible transitions through the Markov process. These results were then used to calculate incremental cost-effectiveness ratios for each comparison in the different patient populations. Given that the model duration was one year, costs and effects were not discounted. The Markov process employed a half cycle correction which meant that patients were attributed their initial health state utility values half way through the first cycle [33].

**Figure 1:**
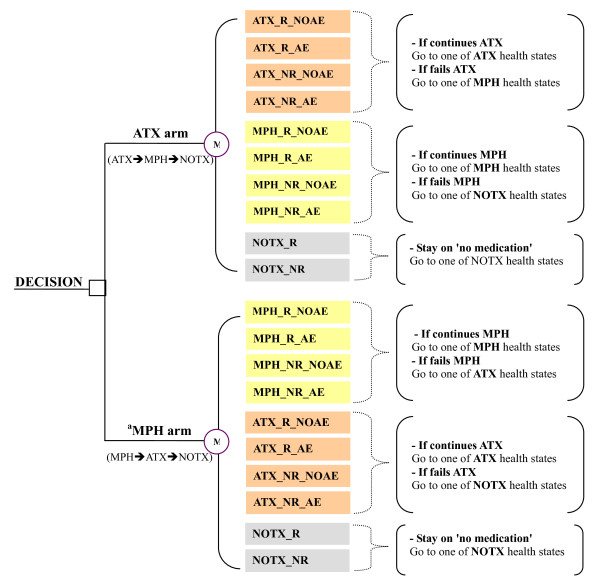
**Structure of the Markov process in population 1**. *Abbreviation*: ATX = atomoxetine; MPH = methylphenidate; NOTX = no medication; R = response; NR = no response; AE = adverse events; NOAE = no adverse events. ^a ^Either IR-MPH or XR-MPH is separately compared to atomoxetine. *Note: The Markov model is similarly structured even when atomoxetine is compared to 'no medication' for those who are ineligible for MPH treatment. In this case, all health states related to MPH are eliminated from the current model.

The Markov process comprised fourteen and six health states for population 1 and for population 2 and 3, respectively. Each health state represented one of a range of possible health outcomes (response and/or occurrence of adverse events) associated with treatment alternatives considered in the economic model. Upon entering the Markov process, patients were distributed into one of four health states associated with atomoxetine or those associated with either MPH (in population 1) or 'no medication (in population 2 and 3). Upon failure of therapy, patients could move through to health states associated with the next treatment options (see Figure 1).

Patients could remain within their resident health state until one of the following events occurred

• The patient discontinues medication due to lack of efficacy: applicable only to patients on an active treatment and in a non-responder health state. The model assumes a maximum of two consecutive non-response cycles. A third non-response cycle results in automatic discontinuation due to lack of efficacy. After discontinuing one medication, the patient will switch immediately to the next alternative in the treatment algorithm to being the next Markov cycle.

• The patient discontinues medication due to a medication-related adverse event and progresses to the next line of therapy.

• An adverse event resolves

• The patient discontinues medication for any other reason: applicable equally to all patients on active treatment. These patients are assumed to stop therapy altogether.

• The patient relapses: applicable only to those patients in a responder health state. A patient who relapses becomes a non-responder in the following Markov cycle.

### Model variables

#### Costs

Costs were estimated from the perspective of the National Health Service in Spain. The economic model considered only the pharmaceutical cost of treatment when comparing medication alternatives, thereby assuming that all non-drug health care costs and indirect costs were equivalent between the treatment groups being compared.

Such an assumption may be considered biased against the active therapies which have the potential to reduce symptoms and consequently, a patient's reliance on health care professionals. Furthermore, the cost of drugs associated with the treatment of medication-related side effects was not considered. Due to the persistence of insomnia, patients treated with stimulant (i.e. MPH) are more likely than patients treated with atomoxetine to require concomitant medications for side effects, indicating that the exclusion of these costs may be biased against atomoxetine.

Cost variables used in the Markov process are presented in Table 1. Most patients with atomoxetine need only a single capsule per day and a single capsule costs €4.34, irrespective of capsule strength. Thus €4.34 was used in the model as the daily cost of atomoxetine. Calculation of the daily cost of MPH was based on the estimated average daily dose taken by patients and the relative use of available pack sizes for each medication according to current market research [34-36]. Unit costs of MPH were derived from data available at the General Spanish Council of Pharmacists (CGCOF) [34-36]. Patients in the model received 30 days of medication per montly cycle.

**Table 1:** Medication costs in the economic model

	**Atomoxetine cost**	**IR-MPH cost**	**XR-MPH cost**
Average daily dose	1 capsule	43.11^a^	43.43^a^
Daily cost of medication	€ 4.34^b^	€ 0.48^c^	€ 2.63^c^
Days on medication per Markov cycle	30	30	30

Cost of medication per Markov cycle	130.20	14.40	78.90

*Abbreviations*: IR-MPH = immediate-release methylphenidate; XR-MPH = extended-release methylphenidate

a. Market research data [34-36]

b. Daily cost of atomoxetine is independent of average daily dose. Cost is based on a cost per capsule, independent of capsule strength [34-36]

c. Daily costs of IR-MPH and XR-MPH based on current costs applied to the average daily dose, weighted by the relative days of therapy of each pack size for each medication [34-36]

#### Health state utility values

Health state utility values for the fourteen possible health states included in the economic model were based on a utility valuation survey of 83 parents of children with ADHD in the UK using standard gamble methodology [37]. Parents were chosen as the most suitable patient proxy respondents on the basis that many ADHD children would be too young to provide reliable responses.

The health state description comprised four domains: 1) descriptors referring to behaviour during different time periods throughout the day; 2) information concerning the child's overall social well-being; 3) attributes regarding medication regimen (e.g. frequency of adminitration); 4) medication-related adverse events. Descriptions for the four domains were derived largely based on systematic review of clinical trials and validated by clinical experts. Further details of the descriptors for the fourteen health states in terms of the four domains can be found elsewhere [37].

The health state corresponding to the atomoxetine 'responder without side effects' had the highest utility value (0.959). Health states corresponding to 'responder without side effects' for XR-MPH and IR-MPH had utility values of 0.930 and 0.913, respectively. The higher utility scores associated with atomoxetine responders are mainly attributable to its better behavioural profiles. Atomoxetine responder health states reflects improved behaviour in the early morning as well as at night [23], whereas stimulant responder health states reflects only for a limited time following administration of medication. For each of the 'no medication' health states, utility values of 0.880 were obtained from the 'child's own health state' as given by a subgroup of 23 parents whose children were not currently receiving medication. Table 2 presents the utiltity scores incorporated in the economic model.

**Table 2:** Utility values derived from the utility valuation survey [37]

**Health State**	**N**	**Mean utility value**	**SD**	**95% CI**
Medication with atomoxetine; responder without side effects	83	0.959	0.077	0.942 – 0.976

Medication with atomoxetine; responder with side effects	83	0.937	0.096	0.916 – 0.958

Medication with atomoxetine; non-responder without side effects	83	0.902	0.133	0.873 – 0.931

Medication with atomoxetine; responder with side effects	83	0.886	0.148	0.854 – 0.918

Medication with IR-MPH; responder without side effects	83	0.913	0.128	0.885 – 0.941

Medication with IR-MPH; responder with side effects	83	0.904	0.137	0.875 – 0.933

Medication with IR-MPH; non-responder without side effects	83	0.889	0.154	0.856 – 0.922

Medication with IR-MPH; responder with side effects	83	0.875	0.164	0.840 – 0.910

Medication with XR-MPH; responder without side effects	83	0.930	0.107	0.907 – 0.953

Medication with XR-MPH; responder with side effects	83	0.912	0.124	0.885 – 0.939

Medication with XR-MPH; non-responder without side effects	83	0.898	0.130	0.870 – 0.926

Medication with XR-MPH; responder with side effects	83	0.884	0.143	0.853 – 0.915

No medication; responder	23	0.880	0.133	0.826 – 0.934

No medication; non-responder	23	0.880	0.133	0.826 – 0.934

*Abbreviations*: IR-MPH = immediate-release methylphenidate; XR-MPH = extended-release methylphenidate; SD = standard deviation

#### Transition probabilities

Transition probabilities used to populate the Markov process and respective data sources are presented in Table 3 and 4. Note that some are data on file and the details of the sources can be found in the UK model paper [27]. In addition, clinical trials directly included in this study for the data synthesis were approved according to local requirements for ethics and/or regulatory approvals for clinical trials.

**Table 3:** Transition probabilities used in the Markov process that do not vary by patient population

			**Probability by treatment**		
		
		**Atomoxetine**	**IR-MPH**	**XR-MPH**	**No medication**
Probability of one or more medication-related adverse events^a^		0.129	0.129	0.129	0.000

Probability that a medication-related adverse event is insomnia^b^		0.000	0.48	0.48	NA

Probability that a medication-related adverse event, which is not insomnia^c^	First 4 cycles	0.473	0.473	0.473	NA
	
	Cycles thereafter	1.000	1.000	1.000	NA

Probability that insomnia will persist from one Markov cycle to the next^d^	First 4 cycles	NA	0.953	0.953	NA
	
	Cycles thereafter	NA	1.000	1.000	NA

Probability that a non-responder discontinues due to lack of efficacy during a Markov cycle^e^		0.0989	0.0989	0.0989	NA

Probability that a patient discontinue due to a medication-related adverse event during a Markov cycle^f^		0.1209	0.1209	0.1209	NA

Probability that a patient discontinues for reasons other than lack of efficacy or a medication-related adverse event during a Markov cycle^e^	First 4 cycles	0.384	0.384	0.384	NA
	
	Cycles thereafter	0.000	0.000	0.000	NA

*Abbreviations*: IR-MPH = immediate-release methylphenidate; XR-MPH = extended-release methylphenidate; NA = Not applicable

a. Probabilities based on *post hoc *analyses of safety data pooled from six randomised placebo-controlled trials of atomoxetine versus placebo [38-40,42] (some are data on file). Assumption of parity between active treatments based on similar *post hoc *analyses of data from a limited open-label direct comparator study [46], supported by data from a double-blind randomised trial of atomoxetine and XR-MPH (data on file) where the proportions of patients experiencing one or more adverse events of any nature were not significantly different between the active treatments. Values are net of the placebo rate, meaning that the 'no medication' probability is zero, by definition.

b. The probability based on the relative risk (0.417) of insomnia (atomoxetine vs IR-MPH), estimated in an indirect meta-analysis of safety data [43], applied to the risk of insomnia for atomoxetine (4.7%) derived from pooled analysis of safety data from six pivotal randomised placebo-controlled trials of atomoxetine [38-40,42] (some are data on file), giving a rate of insomnia for IR-MPH of 4.7/0.417 = 11.27%. The model assumes that insomnia is experienced only as a result of taking medication. Therefore, the probability for placebo is not applicable (i.e. zero) and the probabilities for active treatments are net of the placebo rate (i.e. subtract 5.1%). As a consequence, the model assumes that patients on atomoxetine have no risk of medication-related insomnia. Patients on IR-MPH who experience insomnia will come only from the population who experience one or more adverse events as derived in note 1, therefore, for 'if adverse event, probability that insomnia included' = (11.27-5.1)/12.9 = 48%. Parity is assumed between IR-MPH and XR-MPH [24,26].

c. Probabilities based on temporal course of treatment-emergent adverse events (data on file) where weekly reports from patients treated with atomoxetine over 52 weeks imply that, for most patients, medication-related averse events mainly occur early in the treatment and are likely to resolve within approximately 16 weeks. The probability of 0.473 (0.05^1/4^) for the first four cycles with adverse event(s) reflects a nominal 5% of patients in whom adverse events (that are not insomnia) persists over this duration of the Markov process. The duration of persistence of adverse events (that are not insomnia) is assumed to be similar for each medication.

d. Probabilities based on a survey of six consultant child and adolescent psychiatrists (data on file). Responses suggested that 82.5% of cases of stimulant-related insomnia would persist for more than 16 weeks. The model assumes that patients with stimulant-related insomnia that persists beyond four cycles will continue to have insomnia as long as they remain on treatment. The probabilities of 0.953 (0.824^1/4^) for the first four cycles of the Markov process and 1.000 for cycles thereafter reflect this.

e. Probabilities based on discontinuation rates, regardless of treatment, from data pooled from seven randomised placebo-controlled trials of atomoxetine [38-42] (some are data on file), adjusted for differences between trials with respect to duration of follow-up. Discontinuations due to lack of efficacy were assumed to occur in only the non-responder population. In each case, parity is assumed between the active treatments.

f. Probabilities based on discontinuation rates due to adverse events from data pooled from six pivotal randomised placebo-controlled trials of atomoxetine [38-40,42] (some are data on file), adjusted for differences between trials with respect to duration of follow-up. Discontinuations due to adverse events were assumed to occur only in the population experiencing one or more medication-related adverse events and therefore were net of the placebo rate. In each case, parity is assumed between the active treatments.

**Table 4:** Transition probabilities used in the Markov process that vary by patient population

			**Probability by treatment**		
	
	**Population**	**Atomoxetine**	**IR-MPH**	**XR-MPH**	**No medication**
Probability of response to treatment	1. Stimulant-naïve, not contra-indicated^a^	0.7051	0.7727	0.7727	NA
	2. Stimulant-failed, not contra-indicated^b^	0.6346	NA	NA	0.3731
	3. Stimulant-naïve, contra-indicated^c^	0.6667	NA	NA	0.423

Probability of relapse per 30-day period^d^	1. Stimulant-naïve, non contra-indicated	0.0206	0.0206	0.0206	NA
	2. Stimulant-failed, non contra-indicated	0.0257	NA	NA	0.0447
	3. Stimulant-naïve, contra-indicated	0.0206	NA	NA	0.0387

*Abbreviations*: IR-MPH = immediate-release methylphenidate; XR-MPH = extended-release methylphenidate; NA = not applicable

a. Probabilities of response in stimulant-naïve patients are not contra-indicated are based on a meta-regression analysis [45] of response data from randomised active comparator trials of atomoxetine and MPH [39,46,47] (some are data on file). Assumption of parity between stimulants is based on head-to-head trials of IR-MPH and XR-MPH [24,26].

b. Probabilities of response in MPH-exposed (failed) patients in whom stimulants are not contra-indicated are derived and inferred from responder rates in a crossover trial of atomoxetine and XR-MPH [47]. A probability of response for 'no medication' is derived by applying the relative risk of repsonse for placebo versus atomxetine, drawn from the meta-regression analysis [45].

c. Probabilities of response in stimulant-naïve patients in whom stimulants are contra-indicated are based on responder rates from a randomised placebo-controlled trial of atomoxetine in patients with tics or Tourette's syndrome [41].

d. Probability of relapse = 1 - [(1-C)^(1/E)^], where C = the proportion of patients relapsing and E = approximate total number of follow-up days, derived from a relapse prevention study [48], divided by the approximate number of days per Markov cycle. Parity is assumed between active medications.

Probabilities that did not vary by patient population (Table 3), including probabilities of medication related adverse events and discontinuations from treatment, were derived from placebo-controlled clinical trials for atomoxetine [38-42] (some are data on file) and a published indirect meta-analysis of safety data from randomised placebo-controlled and active comparator studies of atomoxetine and methylphenidate [43].

Medication-related adverse events were defined as any adverse event (i) found to be significant for atomoxetine in a pooled analysis of safety data from six pivotal randomised placebo-controlled trials [38-40,42] (some are data on file) (ii) found to be significant for IR-MPH in a publised quantitative meta-analysis of safety data from randomised controlled trials [44] or (iii) listed as very common (frequency ≥ 10%) for IR-MPH and/or XR-MPH in Summary Product Characteristics [29]. Medication-related adverse events comprised appetite loss, stomach ache, vomiting, somnolence, irritability, dizziness, fatigue, insomnia, headache and nervousness.

Assumptions regarding the persistence of medication-related adverse events were based on long-term treatment data for atomoxetine (data on file), where weekly reports of adverse events, either as a first or repeat occurrence, fell off with time to fairly constant low levels which, in many cases, were considered to be close to the baseline reporting of such adverse events. These data implied that for most patients medication-related side effects mainly occured early on in the treatment and were likely to resolve within approximately 16 weeks.

Data concerning time to resolution for MPH-related adverse events were not available. Since adverse events associated with MPH were mostly considered mild and transient, the model assumed that, with one exception, the time to resolution for MPH-related adverse events was the same as for atomoxetine. The exception to this was stimulant-associated insomnia, which could persist in a proportion of cases. The probability that medication-related insomnia persists in MPH-treated patients was based on responses collected in a survey of consultant child and adolesent pscyhiatrists, all highly experienced in treating children with ADHD (data on file).

Probabilities of response and relapse varied by patient population (Table 4). The evidence base for these variables in each of the populations are described below.

##### Population 1

Probabilities of treatment response in patients with no history of pharmacotherapy use and no contra-indications to stimulants were derived from responder rates estimated in a meta-regression analysis [45] of patient-level data from five randomised active comparator trials of atomoxetine and MPH [39,46,47] (some are data on file).

##### Population 2

Probabilities of response to atomoxetine in patients with a previous MPH treatment failure but no contra-indications to stimulants were derived and inferred from responder rate in the patients treated with atomoxetine after a failure of an initial 6 weeks treatment with XR-MPH in a randomised cross-over study of XR-MPH and atomoxetine [47]. A probability of response on 'no medication' in this population was derived by applying the relative risk of response for placebo versus atomoxetine, drawn from the meta-regression analysis [45].

##### Population 3

The probability of treatment response in patients with no history of pharmacotherapy use and contra-indications to stimulants was derived from responder rate in patients with no history of pharmacotherapy use in a randomised placebo-controlled trial of atomoxetine conducted exclusively in an ADHD patient group who had been co-diagnosed with tic disorder or Tourette's syndrome [41]. The limitation of this, of course, is that patients with tics or Tourette's syndrome constitute a subgroup of, rather than being representative of, the overall stimulant-contraindicated population. However, in the absence of data from a more appropriate patient group, this is best estimate available.

Probabilities of relapse were based on data for stimulant-naïve and stimulant-exposed patients in a placebo-controlled relapse prevention trial of atomoxetine responders [48]. In the absence of comparative data, an assumption of parity was made between relapse rates for all active treatments and also between patients who were contraindicated for stimulants and those who were not.

For all transition probability variables, where applicable, the assumption of parity between IR-MPH and XR-MPH was made based on data published from head-to-head trials of treatments [24,26].

### Sensivity analysis

Extensive sensivity analyses were carried out on cost, utility and transition probability variables.

## Results

The results of the economic model are summarised in Table 5. Overall, treatment with atomoxetine was associated with higher costs and better health outcomes, translated into increased QALYs, when compared to either MPH (both IR-MPH and XR-MPH) or 'no medication'.

**Table 5:** Total costs, QALYs and incremental cost-effectiveness estimated in the economic model by patient population

**Population**	**Cost per patient**		**QALYs per patient**		**Incremental cost per QALY gained**
	**ATX arm**	**Comparator arm**	**ATX arm**	**Comparator arm**	
**Population 1^a ^(comparator: IR-MPH)**	€ 1 047	€ 366	0.930	0.910	€ 34 308
**Population 1^a ^(comparator: XR-MPH)**	€ 1 208	€ 902	0.933	0.920	€ 24 310
**Population 2^b ^(comparator: 'no medication')**	€ 919	€ 0	0.919	0.880	€ 23 820
**Population 3^c ^(comparator: 'no medication')**	€ 969	€ 0	0.922	0.880	€ 23 323

*Abbreviations*: ATX = atomoxetine; IR-MPH = immediate-release methylphenidate; XR-MPH = extended-release methylphenidate; QALY = Quality-adjusted life year

a. Stimulant-naïve patients without contra-indications to stimulants

b. Stimulant-failed patients without contra-indications to stimulants

c. Stimulant-naïve patients with contra-indications to stimulants

For the stimulant-naïve patients without contra-indications to stimulants (population 1), treatment with atomoxetine was associated with additional costs of € 681 compared to IR-MPH and € 306 compared to XR-MPH. For the patients having stimulant-failure experience or contra-indications to stimulants (population 2 and 3), atomoxetine was in principle the only alternative option available. Atomoxetine was thus compared to 'no medication' within these groups of patients. The additional cost of atomoxetine treatment compared to 'no medication' was € 919 in the stimulant-failed patients without contra-indications to stimulants (population 2). Similar cost (€ 969) was incurred as well by atomoxetine treatment in the stimulant-naïve patients but having contra-indications to stimulants (population 3).

Patients who started treatment with atomoxetine experienced slightly less time with adverse events than patients with MPH in population 1, while the duration of response over the 1-year period was similar between the two groups (results not shown). This, together with the higher utility value associated with a response to atomoxetine relative to the MPH or 'no medication', translated into QALY gains for patients treated with atomoxetine. For the stimulant-naïve patients without contra-indications to stimulants (population 1), treatment with atomoxetine was associated with 0.020 and 0.013 additional QALYs gained, when compared to IR-MPH and XR-MPH, respectively. The magnitude of additional QALYs gained associated with atomoxetine, was greater among the patients having stimulant-failure experience or contra-indications to stimulants (population 2 and 3) as 'no medication' (i.e. comparator of atomoxetine in these patient populations) was associated with the lowest utility values of 0.880. The additional QALYs gained associated with atomoxetine treatment was 0.039 and 0.042 in population 2 and 3, respectively.

The incremental cost per QALY gained associated with atomoxetine was consistently lower among the patients having stimulant-failure experience or contra-indications to stimulants (population 2 and 3), in comparison to that of the stimulant naïve patients without contra-indications to stimulant (population 1). The incremental cost per QALY gained of atomoxetine was € 23 820 and € 23 323 in population 2 and 3 respectively while it was € 34 308 and € 24 310 compared to IR-MPH and XR-MPH respectively in population 1. This is an intuitive result because atomoxetine is the most cost-effective in the patient group in which there are no pharmacotherapy alternatives currently available (i.e. in population 2 and 3) and least cost-effective in the treatment naïve patient group for which other pharmacotherapy options are available (i.e. population 1).

In addition, an extensive range of one-way and scenario-based sensitivity analyses were performed for other uncertain model variables and assumptions. In general, the incremental cost per QALY gained in each population was insensitive to changes in key clinical and cost variables (results not shown; the full results of sensitivity analyses are available from the authors upon request). However, the sensitivity analyses show that the utility values of all health states are crucial determinants of the cost-effectiveness of atomoxetine.

Given the importance of the utility values to the results of the economic model, additional sensitivity analyses of the utility values were explored for the stimulant-naïve patients without contra-indications to stimulants (population 1), in which both atomoxetine and MPH were possible treatment options. The additional analysis was to see how the results of the model are affected when differences between utility values of corresponding health states of atomoxetine and those of MPH (either IR-MPH or XR-MPH) are reduced or eliminated.

**Figure 2:**
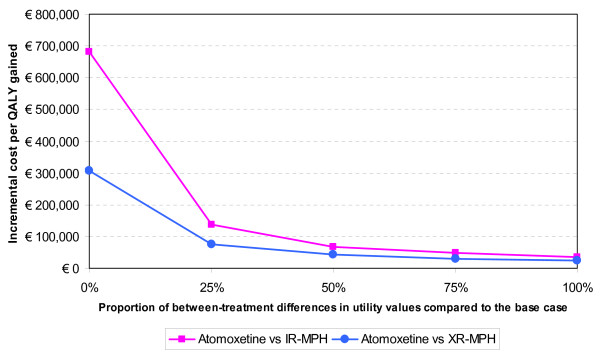
**The ICERs of atomoxetine under varying utility values used in the model in population 1**. *Abbreviations*: IR-MPH = immediate-release methylphenidate; XR-MPH = extended-release methylphenidate; QALY = Quality of life years.

The incremental cost per QALY gained associated with atomoxetine was € 34 308 (compared to IR-MPH) and € 24 310 (compared to XR-MPH) for the base case analysis. When differences in the utility values between corresponding health states of different treatments were reduced by 25% by increasing the base case utility values of MPH, the incremental cost per QALY gained became € 48 643 and € 30 685 (see Figure 2). It again increased to € 68 101 and € 43 835 when differences in utility values were reduced by 50%. Finally, when differences in utility values are eliminated (i.e. 100% reduction), the incremental cost per QALY ratios went up dramatically.

This sensitivity analysis shows that when differences in utility values between treatment groups are removed the incremental cost per QALY gained of atomoxetine rises to unacceptable levels. However, the modest increase in the cost per QALY when differences are reduced by up to 50% and the sound methodology used to derived the utility values [32] serves to minimise the uncertainty surrounding the utility values and thus maximise the reliability of the base case model results.

## Discussion

This study sought to apply pharmacoeconomic modelling techniques to the process of informing the selection of a cost effective treatment for children with ADHD in Spain. Atomoxetine, a newly introduced treatment option in the Spanish market, was compared to methylphenidate, which had been the only approved medication available for ADHD children in Spain. In addition, atomoxetine was compared to 'no medication' among those who were ineligible for methylphenidate treatment (i.e. patients having a history of stimulant-treatment failure or co-morbidities contra-indicated to stimulants). The results of the economic model showed that atomoxetine is associated with better outcomes in terms of QALYs over a 1-year time horizon, compared to methylphenidate (both IR-MPH and XR-MPH) as well as 'no medication'. Although response rate was found to be equal or higher for methylphenidate, patients responding to atomoxetine appeared to experience a more stable and longer-lasting response (throughout a night till the following early morning) [23] than those patients responding to methylphenidate. In addition, parents of the ADHD children tended to prefer nonstimulants to stimulants when the rest is the same otherwise. The nature of such response with atomoxetine and parent preferences for nonstimulants, which were reflected in the utility value survey conducted by Secnik and colleagues [37], led to higher utility values associated with atomoxetine treatment and thereby a greater number of QALYs overall in the context of the economic evaluation.

Overall, the incremental costs per QALYs gained of atomoxetine were between € 23 323 and € 34 308, depending on the patient population. The incremental costs per QALYs gained were consistently lower when atomoxetine was compared to 'no medication', although 'no medication' option was associated with 'zero costs'. This clearly shows that introducing atomoxetine in Spain is beneficial at least for those who are ineligible for methylphenidate treatment as they do not have any treatment alternatives otherwise. Even when compared with methylphenidate (in particular, XR-MPH), atomoxetine as first line therapy was found to be a cost-effective strategy in the treatment of ADHD in Spain [49].

The clinical inputs to the economic model were primarily based on head-to-head randomised clinical trial evidence. Sensitivity analyses confirmed that base case results were likely to be insensitive to changes in input parameters, with the exception of utility values. The utility values appeared to be a key component in determining the cost-effectiveness of atomoxetine. However, these utility values were obtained from a robust utility valuation study of ADHD health states [37] that involved parents of children with ADHD as the respondent population and employed standard gamble methodology. In order to minimise any uncertainty or bias surrounding the utility values, the health states descriptors used in the study interviews were derived largely based on data from randomised clinical trials and validated by clinical experts [37]. Nevertheless, caution is required when interpreting these utility values since there is no large trial to confirm and validate the health states descriptors. Furthermore, there have been concerns over the use of utilities (and therefore QALYs) for the paediatric population [50]. While ADHD children tend to underestimate their 'disease-specific problems', the validity of utilities elicited with parent proxies has not been fully understood yet [28]. The validation of these utility values is necessary when data become available from sufficiently powered randomised controlled trials.

Several limitations of this study are worth noting. Firstly, our study only considered the drug treatments of ADHD. However, behavioural treatment represents 'a therapeutic mainstay in most European countries' [22,51]. In the recent cost-effectiveness analysis based on the MTA data [52], behavioural treatment was found to be more cost effective than intensive medication management (mainly stimulants) in ADHD children with co-morbid conditions whereas the opposite was true in pure ADHD children (i.e. no co-morbid conditions). The study then suggested behavioural treatment with or without medication as the most cost-effective choice for co-morbid ADHD population. While behavioural treatment is certainly of value, further research to examine the cost-effectiveness of ADHD treatments including nonstimulant atomoxetine remains worthwhile and valuable since stimulants are often contra-indicated for co-morbid conditions. Secondly, patient group involved in the utility survey were those living in the UK and thus their response may differ from those living in Spain. However, as there were no available utility values of treatments for ADHD children in Spain, those utility values used here were the best available data. Thirdly, the perspective of this analysis was that of the National Health Service in Spain and thus only direct medical costs were considered in the model. The exclusion of indirect costs however may be biased against active treatments as effective treatments are likely to decrease these costs. Therefore the estimates of cost-effectiveness of atomoxetine compared to 'no medication' option can be considered conservative. Fourthly, it could be argued that a longer timeframe may be desirable to take into account longer-term differences in costs and adverse effects of treatments. However, any omissions due to the shorter timeframe are likely to be again conservative in that they bias the model generally against the active therapies and more specifically against atomoxetine. For example, the model does not allow for the pattern of care of ADHD patients to change according to response to active therapy. This means the omission of non-drug costs within the model are assumed to be the same across all disease health states. Finally, in an ideal situation it would be preferable to have utility scores estimated from the patient perspective. However, the use of parents of ADHD children as patient proxies is seen to provide the best practical alternative.

## Conclusion

In general, the results of this study showed atomoxetine to be within the bounds of reasonable cost-effectiveness for Spain. In comparison with methylphenidate, atomoxetine as first line therapy appeared as a cost-effective strategy. The additional value offered by atomoxetine was even clearer for those who have no alternative treatment options otherwise. The results of this analysis are considered to be robust, having been based on the best available clinical evidence, expert opinion and a rigorously conducted utility valuation study of ADHD-related health states.

## Competing interests

This study was sponsored by Eli Lilly and company. Jihyung Hong is currently doing her PhD at LSE and also working as a consultant for Eli Lilly and company. Tatiana Dilla and Jorge Arellano are employees of Eli Lilly and Company.

## Authors' contributions

JH reconstructed the UK economic model for adaptation to the Spanish context, and also wrote this manuscript. TD gave contribution to conception and design and in the critical review of the manuscript. JA, who was one of the contributors for the original UK model, also contributed to a crucial review of this manuscript. All authors read and approved the final manuscript.

## Pre-publication history

The pre-publication history for this paper can be accessed here:


